# Changes in blood lymphocyte numbers with age in vivo and their association with the levels of cytokines/cytokine receptors

**DOI:** 10.1186/s12979-016-0079-7

**Published:** 2016-08-18

**Authors:** Yun Lin, Jiewan Kim, E. Jeffrey Metter, Huy Nguyen, Thai Truong, Ana Lustig, Luigi Ferrucci, Nan-ping Weng

**Affiliations:** 1 Laboratory of Molecular Biology & Immunology, National Institute on Aging, 251 Bayview Blvd., Baltimore, MD 21224 USA; 2 Translational Gerontology Branch, National Institute on Aging, National Institutes of Health, Baltimore, MD 21224 USA; 3 Department of Neurology, University of Tennessee Health Science Center, Memphis, TN 38111 USA

**Keywords:** Aging, Human, Peripheral blood, Lymphocytes, CD4 and CD8 T cell, B cell, NK cell, CMV

## Abstract

**Background:**

Alterations in the number and composition of lymphocytes and their subsets in blood are considered a hallmark of immune system aging. However, it is unknown whether the rates of change of lymphocytes are stable or change with age, or whether the inter-individual variations of lymphocyte composition are stable over time or undergo different rates of change at different ages. Here, we report a longitudinal analysis of T- and B-cells and their subsets, and NK cells in the blood of 165 subjects aged from 24 to 90 years, with each subject assessed at baseline and an average of 5.6 years follow-up.

**Results:**

The rates of change of T-(CD4^+^ and CD8^+^) and B-cells, and NK cells were relative stable throughout the adult life. A great degree of individual variations in numbers of lymphocytes and their subsets and in the rates of their changes with age was observed. Among them, CD4^+^ T cells exhibited the highest degree of individual variation followed by NK cells, CD8^+^ T cells, and B cells. Different types of lymphocytes had distinct trends in their rates of change which did not appear to be influenced by CMV infection. Finally, the rates of CD4^+^, CD8^+^ T cells, naive CD4^+^ and naïve CD8^+^ T cells were closely positively correlated.

**Conclusion:**

Our findings provide evidence that the age-associated changes in circulating lymphocytes were at relative stable rates in vivo in a highly individualized manner and the levels of selected cytokines/cytokine receptors in serum might influence these age-associated changes of lymphocytes in circulation.

**Electronic supplementary material:**

The online version of this article (doi:10.1186/s12979-016-0079-7) contains supplementary material, which is available to authorized users.

## Background

Decline in immune function with age is viewed as a fundamental problem for the increased risk of age-associated diseases or disabilities [1–3]. One of the hallmark changes in the immune system with age is the alteration of the number and composition of different types of lymphocytes in the circulation. In older individuals, the numbers of CD4^**+**^ and CD8^**+**^ T cells and B cells are reduced whereas the numbers of NK cells are increased as compared to younger individuals [4, 5]. At the subset level, decreases of naïve T and B cells and increases of memory T and B cells also occurs with aging [6–11]. Such changes may reflect a combination of reduced production of naïve lymphocytes and the accumulation of memory lymphocytes as the results of the reduced overall production of lymphocytes and of the host-environment interaction over time. Despite the overall trend of age-associated changes, striking variations in the numbers of lymphocytes exist between individuals. It is currently unknown whether the observed variations are due to stable characteristics that are maintained over time or whether different subjects have different rates of change with aging.

In the T cell compartment, age associated reduction of CD4^**+**^ and CD8^**+**^ T cells as a percentage of peripheral blood mononuclear cells (PBMCs) as well as absolute numbers (cell/μl) in blood have been reported [5, 12, 13]. Within the T cell subsets, in addition to reduced naïve and increased memory CD4^**+**^ and CD8^**+**^ T cells with age, studies have shown that CD4^**+**^ regulatory T cells and CD8^**+**^CD28^-^ T cells increase with age [14–16]. Thymic involution is the single most critical contributor of reduction in naïve T cells with age [17] whereas cumulative encounters with antigens over time is the force driving the increase memory T cells [18], CD8^**+**^CD28^-^ T cells [16], as well as Tregs [19]. A similar decrease of naïve and increase of memory B cells also occurs in the B cell compartment but the magnitude of change is not as profound as what is observed in T cells [6, 20]. Interestingly, natural killer cells (NK cells) are the only lymphoid linage cells that increase with aging [4, 21, 22]. However, the cytotoxic activity of NK cells appears to be reduced with age and thus the increase in NK cell number may be interpreted as compensatory. Some of the alterations in lymphocyte composition are considered biomarkers of immunosenenscence (ratio of CD4/CD8, increase of CD28^-^ T cells, and increase of NK cells) because they are associated with mortality in elderly [23, 24].

Information regarding age-related changes in lymphocyte composition in humans is mostly derived from cross-sectional studies. This approach may be biased by selective mortality or participation attrition and, in addition, lack the time dimension necessary to dissect cross-sectional and longitudinal effects. Data from longitudinal studies should allow for the determination of whether changes in lymphocyte compositions occur at a constant rate or are non-linear over time and whether there are detectable causes of these changes.

Here, we conducted a longitudinal analysis of CD4^**+**^ and CD8^**+**^ T cells, B cells and their subsets, and NK cells in 165 participants from the Baltimore Longitudinal Study of Aging (BLSA) (https://www.blsa.nih.gov/) assessed at baseline and, on average, after a 5-year follow-up. We analyzed the rates of changes and explored potential causes of variations and correlation among these changes and with CMV infection. Our findings provide detailed longitudinal rates of changes in lymphocytes and their subsets with aging in a relatively healthy population dispersed over a relatively wide age-range.

## Methods

### Study design and participants

We performed a longitudinal study of T- and B-cells and their subsets, and NK cells in peripheral blood of 165 BLSA participants at first visit and 5-year follow-up under the NIH IRB- approved protocol (GRC98-12-28-01) and performed in accordance with the Declaration of Helsinki. Demographic characterization of these participants was summarized in Additional file 1: Table S1. At each visit, blood cell counts were measured by standard complete blood cell counts by Coulter Counter and PBMCs were isolated from 50 mL blood drawn from participants under fasting condition and cryopreserved in liquid nitrogen. Two to five cryopreserved PBMCs from each subject with an average of 5.6 years apart were used in the experiments. PBMCs from all time points were thawed and counted on the day of the experiment. The recovery of frozen PBMC was 77 % ± 0.3 % (mean ± SEM). Complete blood cell counts was combined with flow cytometry analysis (see gating strategy in Additional file 1: Figure S1) to obtain the cell count and to estimate rate changes for different cell populations.

### Analysis by flow cytometry

Antibodies used for flow cytometry analysis included: CD2-Tri-Color (TC); CD4-Phycoerythrin (PE) and CD4Allophycocyanin (APC); CD28-Fluorescein Isothiocyanate (FITC); CD8-TC; CD19-APC; CD45RA-APC; CD16-FITC from Life Technologies (Grand Island, NY); CD14-PE; CD27-PE; and IgM-FITC from BD Biosciences (San Jose, CA). Freshly thawed PBMCs from each visit were stained with three to five antibodies: T cells (CD2, CD4, CD8, CD45RA, and CD28); B cells (CD19, IgM, and CD27); NK cells (CD16). The data were collected on a BD FACSCalibur or BD FACSCanto II, and analyzed by Cell-Quest (BD Biosciences) and FlowJo. The gating strategies were presented in Additional file 1: Figure S1.

### Measurement of selected biomarkers and CMV IgG

Fasting blood was collected at each visit for measurement of complete blood cell count and other routine blood chemistry using the standard method under the BLSA protocol. Sera were isolated from blood and stored in a -80 °C freezer prior to cytokine (IL) measurement using a custom-made multiplex assay (BioRad Luminex Assays) according to the manufacturer’s instruction. CMV IgG was measured from sera of 120 subjects (117 of them had two time points and 3 had single time points) using the ELISA kit (Abcam, # ab108639) according to the manufacturer’s instruction.

### Statistical analysis

Figures were plotted as scatterplots with a linear regression line. The regression lines for rate and percentage rate of change by age were analyzed using linear regression. Regressions of number of cells and percent of cells were tested using mixed effects linear regression on age with a random effect for subject to address the within-subject correlation with the repeated measurements. The inclusion of the time difference between the measurements did not affect the assessment. For the regression models, all tests were performed with a *p* < 0.05. Correlations were calculated between pairs of variables, and after adjusting by FDR [25], p value less or equal to 0.005 is considered as significant. To address the question whether rapid rates of change are present across more cell types than expected, rates for each cell type were dichotomized and summed to identify subjects in the tertile with the greatest rate of cell loss (except for NK rates which were the highest tertile). The summed score was compared to the expected binomial distribution by chi-squared test. Assuming a binomial distribution, the probability coefficient was estimated from the data using the Bayesian modeling program rstan (http://mc-stan.org/rstan.html). A single sample proportion test was used to test whether the proportion of subject with summed score of 4 or more was greater in the data as compared to the expected summed score of 4 or more from a binomial distribution with probability of 0.33.

All statistical analyses were done using R version 2.12.1 (http://www.r-project.org).

## Results

### Changes of CD4^+^ T cells and their subsets in peripheral blood with age in vivo

**Fig. 1:**
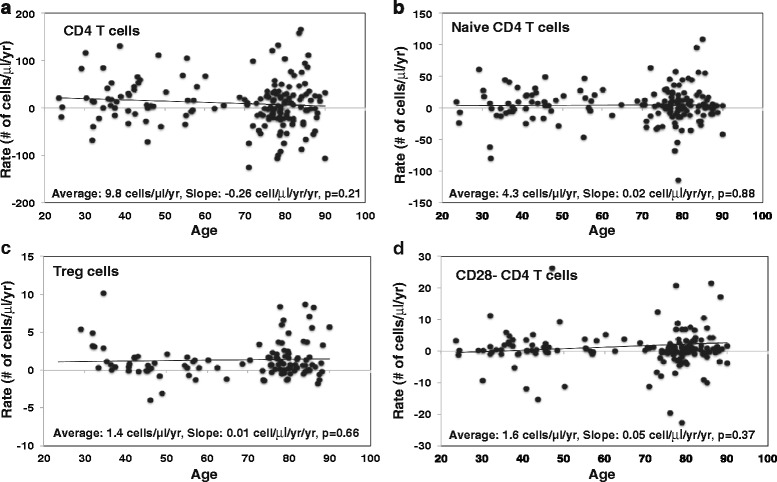
Rate of CD4^+^ T cells and subsets change with age in vivo. **a** Rate of CD4^+^ T cells in peripheral blood in number of cells per μl blood. The rate of CD4^+^ T cells was calculated based on flow cytometry analysis using the gating strategy described in (Additional file 1: Figure S1) and lymphocyte counts from complete blood counts. The linear regressions rate over time are -0.26 cell/μl/year (*N* = 165). **b** Rate of naïve CD4^+^ T cells in CD4^+^ T cells in number of cells per μl blood. The rate of naïve CD4^+^ T cell in cell/μl/year was based on flow cytometry analysis and lymphocyte counts from complete blood counts (*N* = 158). **c** Rate of regulatory CD4 T (Treg) cells in peripheral blood (*N* = 112). Treg was defined by expression of CD25 and Foxp3. **d** Rate of CD4^+^CD28^-^ T cells in peripheral blood (*N* = 160). P values were calculated by linear regression in this and subsequent figures

In agreement with previous reports, at baseline older age was associated with slightly lower number of CD4^**+**^ T (Additional file 1: Figure S2a) [26, 27]. Rates of change of CD4^**+**^ T cell, reported as cell number per μl blood per year, were quite heterogeneous across study subjects (ranging from -120 to +170 cells/μl/yr and an average of 9.8 cells/μl/yr) and were relatively stable at different ages (Fig. 1a). Next, we examined rates of change in three subsets of CD4^**+**^ T cells, including naïve (CD45RA^+^CD28^+^), Treg (CD25^+^Foxp3^+^), and CD28^-^ cells. Similar to previous reports, older age was associated with fewer naïve CD4^**+**^ T cells (Additional file 1: Figure S2b) [28, 29]. Similarly to the total CD4^**+**^ T cells, there were remarkable individual variability in rates of change in naïve CD4^**+**^ T cells (ranging from -80 to +108 cells/μl/yr and average 4.3 cells/μl/yr) but the average trend of change with age was surprisingly flat, suggesting that naïve phenotype CD4^**+**^ T cells were well maintained throughout the adult life span in the absence of apparent new genesis from the thymus (Fig. 1b). In agreement with previous reports [14, 30], Treg (CD4^+^CD25^+^FOXP3^+^) tended to be more numerous in older than in younger individuals (Additional file 1: Figure S2c). However, the degree of individual variations in the rates of change in Treg (ranging from -4 to +10 cells/μl/yr and average 1.4 cells/μl/yr) was smaller than that of naïve cells but similarly stable in the trend of change with age (Fig. 1c). We observed a similar age-related increase of CD4^**+**^CD28^-^ T cells from ages 20 to 90 (Additional file 1: Figure S2d). Again, the rate of change in CD4^**+**^CD28^-^ T cells showed individual variations and the trend of rate of change with age was relatively stable (ranging from -23 to +60 cells/μl/yr and average 1.6 cells/μl/yr) (Fig. 1d). Together, these results showed a great degree of individual variation in the rates of change in CD4^**+**^ T cells and their subsets among study subjects. Overall there was a rather stable trend in the rates of changes in CD4^**+**^ T cells and their subsets over the adult life time.

### Changes in CD8^+^ T cells and their subsets in peripheral blood with age in vivo

**Fig. 2:**
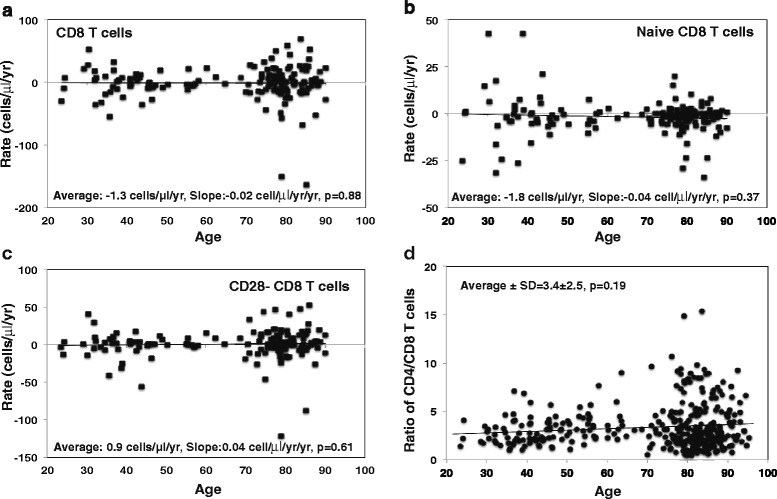
Rate of CD8^+^ T cells and subsets change with age in vivo. **a** Rate of CD8^+^ T cells in peripheral blood in number of cells per μl blood (*N* = 162). The rate of CD8^+^ T cells was calculated based on flow cytometry analysis using the gating strategy described in (Additional file 1: Figure S1) and lymphocyte counts from complete blood counts. **b** Rate of naïve CD8^+^ T cells in CD8^+^ T cell in cell number per μl blood (*N* = 159). Naïve CD8^+^ T cell were defined by CD45RA^+^/CD28^+^. **c** Rate of CD8^+^CD28^-^ T cells in CD8^+^ T cell in cell number per μl blood (*N* = 162). **d** Ratio of CD4^+^/CD8^+^ T cells (number of cells/μl) as a function of age (*N* = 162). P values were calculated by linear regression in this and subsequent figures

In CD8^**+**^ T cells, we observed a similar age related reduction in the number of cells/μl blood in our study cohort of cross-sectional analysis as previously reported [31–33] (Additional file 1: Figure S3a). The rate of change in CD8^**+**^ T cells (ranging from -163 to +69 cells/μl/yr and average -1.3 cells/μl/yr) showed a comparable degree of variation as observed in CD4^**+**^ T cells and the overall rate of change of CD8^**+**^ T cells was remarkably stable throughout the adult lifetime (Fig. 2a). Decrease of naïve CD8^**+**^ T cells in blood with age was observed in this study cohort (Additional file 1: Figure S3b) as well as in previous reports [5, 12, 13]. The rates of naïve CD8^**+**^ T cells (ranging from -34 to +43 cells/μl/yr and average -1.8 cells/μl/yr) also displayed a high degree of individual variations with no significant change of the average rates at different ages (Fig. 2b). The increase of CD8^**+**^CD28^-^ T cells with age is considered as a signature of T cell aging and was observed in this study (Additional file 1: Figure S3c). Interestingly, while the rates of change with age of CD8^**+**^CD28^-^ T cells showed individual variations (ranging from -121 to +53 cells/μl/yr and average 0.9 cells/μl/yr), the average trend was not significantly changed with age, suggesting that the increase of CD8^**+**^CD28^-^ T cells was accumulated through a relatively constant rate over the adult lifetime (Fig. 2c). Collectively, the individual variations in the rates of changes in CD8^**+**^ T cells and their subsets were similar to those of CD4^**+**^ T cells; the overall trend of the rates of CD8^**+**^ T cells and their subsets were largely stable over the adult life. Previous studies suggest that a reduction of the CD4/CD8 ratio with increasing age is an “Immune Risk Profile (IRP)” [23, 24, 34, 35]. Here, we evaluated whether the CD4^**+**^/CD8^**+**^ ratio reduced with age along with the number of cells in the blood, and found that the average ratios of CD4/CD8 T cells were relatively stable with age (mean ± SD = 3.4 ± 2.5) (Fig. 2d). Thus, a reduction in CD4^**+**^/CD8^**+**^ ratio was not observed as a general trend of age in this study cohort.

### Changes in B cells and their subsets in peripheral blood with age in vivo

**Fig. 3:**
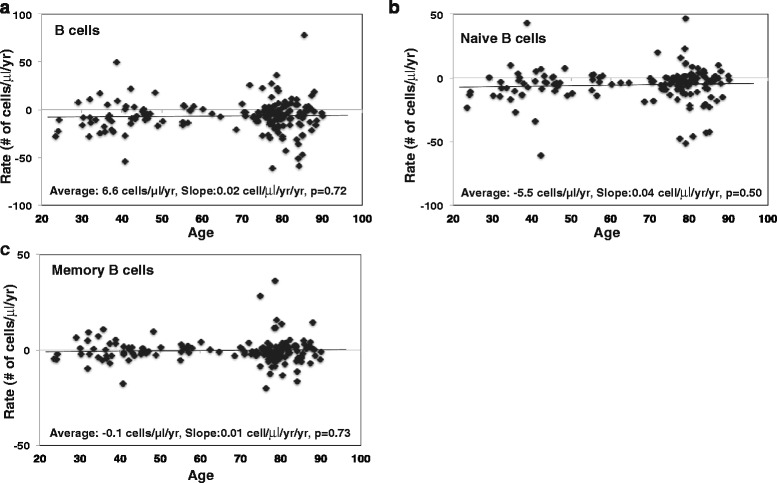
Rate of B cells and subsets change with age in vivo. **a** Rate of B cells in peripheral blood in cell number per μl blood. The rate of B cell was calculated based on flow cytometry analysis using the gating strategy described in (Additional file 1: Figure S1) and lymphocyte counts from complete blood (*N* = 162). **b** Rate of naïve B cells in B cell in cell number per μl blood (*N* = 218). Naïve B cells were defined by CD19^+^IgM^+^CD27^-^. **c** Rate of memory B cells in B cell in cell number per μl blood (*N* = 162). Memory B cells were defined by CD19^+^CD27^+^

B cells were defined based by the expression of CD19 and naïve and memory B cells were defined based by CD19^+^IgM^+^CD27^-^ and CD19^+^CD27^+^, respectively (Additional file 1: Figure S1). We observed a reduction of total B cells as well as naïve B cells with age in our study cohort from the cross-sectional analysis (Additional file 1: Figure S4a-b), and a slight increase in memory B cells with age (Additional file 1: Figure S4c). The average rate of change in B cells was -6.6 cells/μl/yr, and the trend of the rate did not differ across the adult age span (Fig. 3a). Similarly, the average rate of change in naïve B cells was -5.5 cells/μl/yr, and the trend of the rate across the adult age span was flat (Fig. 3b). The average rate of change of memory B cells was -0.1 cells/μl/yr across the age span (Fig. 3c). Overall, these findings showed that the average loss of B cells and naïve B cells with age in vivo did not alter with the subject’s age.

### Change of NK cell and its subsets in peripheral blood with age in vivo

**Fig. 4:**
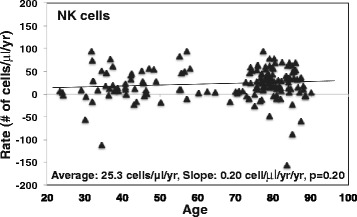
Rate of NK cells changes with age in vivo. The rate of NK cells was calculated based on flow cytometry analysis using the gating strategy described in (Additional file 1: Figure S1) and lymphocyte counts from complete blood counts (*N* = 162)

Increase of NK cells in peripheral blood with aging has been reported [4, 21, 36] and was also observed in this study (Additional file 1: Figure S5). Here, we analyzed the major population of NK cells defined by CD14^-^CD16^+^ (Additional file 1: Figure S1), which is composed of around 90 % of NK cells (Le Garff-Tavernier *et al.*, 2011). The rate of change in NK cells displayed wide individual variation among the study subjects (from -180 to 100 cells/μl/yr) (Fig. 4). Among the average rates of T (CD4 and CD8) and B cells, the average rate of NK cells of all subjects was the highest (25.3 cells/μl/yr) with no change in the rate with age (Fig. 4).

### Correlation between changes among different types of lymphocytes and selected cytokines and soluble cytokine receptors, and antibodies against cytomegalovirus (CMV) in blood

**Fig. 5:**
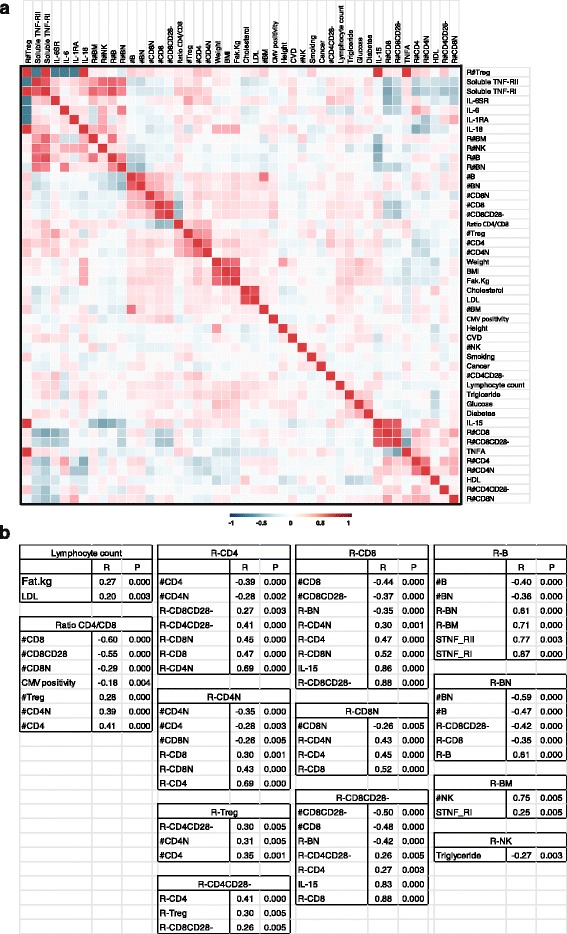
Correlation of rate changes and cytokines/soluble receptor levels in sera. **a** Correlation coefficient among the rate changes of lymphocytes and selected cytokines/their receptors. The correlation coefficients between pair comparison were analyzed with adjustment for age, and presented as a clustered heat map. # refers to the cell counts (x10^3^/μl) and R# refers to the rate of changes used cell counts (cell counts/year). **b** Significant correlations were presented with correlation coefficient (R) and p values. After adjusting by FDR, p value is significant less or equal to 0.005

To determine whether the rates of change in subsets of lymphocytes correlated with each other and with some physiological measurements such as levels of cytokines and soluble cytokine receptors in blood and other physiological parameters, we compared the rate of change of each type of lymphocyte subset with the percents and counts of lymphocytes and a panel of cytokines and physiological parameters (Additional file 1: Table S2) using Pearson’s partial correlation coefficient adjusted for age (Fig. 5). Lymphocyte count was positively correlated with body fat and LDL in sera (Fig. 5). The rate of change in CD4^+^ T cells showed a positive correlation with the rates of change for naïve CD4^+^ and naïve CD8^+^ T cells, total CD8^+^ T cells, CD4^+^CD28^-^ cells and CD8^+^CD28^-^ cells, but a negative correlation with numbers of CD4^+^ cells and naïve CD4^+^ cells (Fig. 5).

The rate of naïve CD4^+^ T cells was positively correlated with the rates of change of CD4^+^, CD8^+^ and naïve CD8^+^ T cells but negatively correlated with numbers of CD4^+^ and naïve CD4^+^, and naïve CD8^+^ T cells (Fig. 5). The rate of Treg cells was positively correlated with numbers of CD4^+^ and naïve CD4^+^ T cells, and rate of CD4^+^CD28^-^ cells (Fig. 5). To determine whether there is a tendency for subjects who show rapid rates of change in one cell type to show similar changes in other cell types, we dichotomized the rates for each cell type to identify subjects in the tertile with the greatest rate of cell loss (except for NK rates which were the highest tertile) and the seven dichotomized rates were summed (total score 0 to 7). If the cell rates are independent, the distribution should be binomial with probability 0.33, which was not the case by chi-squared test (*p* < 0.0001). The probability which fits the summed score was 0.38 that is significantly different that the 0.33 used in creating the score (*p* < 0.02). If the cell rates occur together, the expectation would be for an excessive proportion of subjects to have high summed scores. The expected probability of a score of 4 or more is 0.17, while the observed proportion is 0.23 (*p* = 0.03). The high proportion of high counts argues that there is evidence that rapid rates tend to co-occur among cell types far more often than would be expected by chance (i.e. being independent). Finally, the rate of CD4^+^CD28^-^ cells was positively correlated with the rates of CD4^+^, Treg and CD8^+^CD28^-^ T cells (Fig. 5).

The rate of CD8^+^ T cells showed a positive correlation with the rates of CD8^+^CD28^-^, naïve CD8^+^, total CD4^+^, naïve CD4^+^ T cells and serum level of IL-15 and a negative correlation with number of CD8^+^ T cells, CD8^+^CD28^-^ T cells, and rate of naïve B cells (Fig. 5). The rate of change of naïve CD8^+^ T cells was positively correlated with rates of change of CD4^+^, CD8^+^, and naïve CD4^+^ T cells but negatively correlated with the numbers of naïve CD8^+^ T cells (Fig. 5). Finally, the rate of change of CD8^+^CD28^-^ T cells was positively correlated with the rates of CD8^+^, CD4^+^, CD4^+^CD28^-^ and serum level of IL-15 but negatively corrected with numbers of CD8^+^ CD8^+^CD28^-^ T cells, and rate of naïve B cells (Fig. 5). The ratio of CD4^+^/CD8^+^ T cell number was positively correlated with numbers of CD4^+^, Treg, and naïve CD4^+^ but negatively numbers of CD8^+^, CD8^+^CD28^-^, and naïve CD8 T cells, and CMV seropositivity defined by the presence of anti-CMV IgG antibodies in sera (Fig. 5). The association CMV seropositivity with the low ratio of CD4^+^/CD8^+^ found here is in agreement with the previous report [37].

The rate of B cells was positively correlated with serum levels of soluble TNFRI and TNFRII, and rates of naïve and memory B cells but was negatively correlated with the numbers of B cells and naïve B cells (Fig. 5). The rate of naïve B cells was positively correlated with rate of B cells but negatively correlated with numbers of naïve B, naïve B cells, rates of CD8^+^CD28^-^ and CD8^+^ T cells (Fig. 5). The rate of change of memory B cells was positively correlated with serum levels of soluble TNFRI and numbers of NK cells (Fig. 5). Finally, the rate of change of NK cells was negatively correlated with the serum level of triglyceride (Fig. 5).

## Discussion

Limited information is available regarding the trajectories of in vivo aging of immune cells in humans. As lymphocyte numbers in peripheral blood exhibit a great degree of individual variation, it is unclear whether inter-individuals differences are due to individual’s characteristics that remain stable with aging or result from the different rates of changes in different types of lymphocytes in across individuals. Our longitudinal analysis showed that the rates of changes of all four major types of lymphocytes (CD4^+^, CD8^+^, B, and NK cells) were also highly individualized. The average rates of change of all four major types of lymphocytes were quite stable in the study subjects across 70 years of adult life. It will be necessary to further examine these changes in a longer time follow-up such as 10 or 20 years to understand the contributing genetic and environmental factors that determine the individual variation. Such a study will require long-term commitment and resources but the yield will have the details and resolution needed to better understand these age-associated changes within the immune system.

One of the most prominent signs of an immune system aging is a significant reduction of naïve lymphocytes in blood. The reduction of naïve lymphocytes occurs continuously with advancing age, which has been mainly attributed to a reduction of thymic output after puberty and imperfect peripheral maintenance. However, it is unknown whether the loss of naïve lymphocytes occurs at a constant or increasing rate with age. Our longitudinal analysis showed that the trends of rates of change of naïve CD4^**+**^ and CD8^**+**^ T cell and naïve B cells over 70 years of adult life were remarkably flat, indicating that the continuous reduction of naïve T and B cells in blood is due to a cumulative effect during aging. This conclusion is further supported by study using ^2^H_2_O labeling showed similar dynamics of lymphocytes between young and elderly subjects [38]. In contrast to naïve lymphocytes, CD28^-^ T cells (both CD4^+^ and CD8^+^) and NK cells increase in blood with age. Interestingly, we observed that the trends of the rates of change of CD4^+^CD28^-^ T cells and NK cells (Figs. 1c and 4a) but not CD8^+^CD28^-^ T cells (Fig. 2c) also slightly increase with age. This suggests that age-associated increase of CD4^+^CD28^-^ T cells and NK cells with age is not only accumulated over time but also increased in pace with age.

A reduced CD4^**+**^/CD8^**+**^ ratio is considered as one of the immune risk phenotypes (IRP) related to increased morbidity and mortality in elderly [23, 24, 34, 35]. In agreement with previous finding [37], we also found that the ratio of CD4/CD8 T cells is negatively correlated with CMV seropositivity and the trend of the CD4^**+**^/CD8^**+**^ ratio was not substantially changed with age (Fig. 2e). Collectively, these findings suggest that a reduction in the CD4^**+**^/CD8^**+**^ ratio is not a general change with age in this study cohort but is more obvious to the CMV infected old subjects.

Correlational analysis of the rates of change of lymphocytes and their subsets with some selected cytokines and soluble cytokine receptors revealed some unexpected findings. We found that the rates of CD8^+^ and CD8^+^ CD28^-^T cells were positively correlated with levels of serum IL-15. In contrast to T cells, rates of B cells and memory B cells were positively correlated with soluble TNF-RI. IL-15 is a critical growth factor for CD8^+^ T cells [39] and TNF alpha can induce B cell proliferation [40], their positive correlations suggest that cytokine mediated peripheral expansion is an influencing factor of the rate of changes.

We have applied 11 cell lineage/differentiation makers in analyzing 4 major lymphocyte populations in blood. Although using frozen PBMCs in this study prevents selection of those temperature sensitive markers such as CD62L and CCR7, there are more differentiation markers that can be used to improve the resolution and precision of age-associated changes in these lymphocyte subsets. In particular, applying IgD and IgG staining could further separate naïve and memory B cell subpopulations as some of those subpopulations display more obvious age-related changes [41–43]. Therefore, further study is warranted to precisely assess the age-associated change of lymphocyte composition and function in vivo.

## Conclusion

In conclusion, our findings showed that the rates of changes in T (CD4^**+**^ and CD8^**+**^), B, NK cells and their subsets are highly individualized, exhibiting a wide range regardless of the subject’s age and the average trends of the rates of changes were relatively flat over 70 years of adult life. Unexpectedly, we observed significant associations of the serum levels of certain cytokines and cytokine receptors with the rates of change in selected lymphocytes and their subjects. Collectively, our findings provide much needed information of the in vivo changes of lymphocyte compositions and numbers in blood with age, the interrelationship among different type of lymphocytes and their subsets, and potential contributions of serum cytokines/cytokine receptors in these age-associated changes. Future study with a longer time span, samples with multiple time points and assessment with more differentiation markers will further enhance our understanding of how and when the alterations of lymphocyte type, composition, and number occur, and will be essential to better guide clinical interventions such as vaccination with improved precision and efficacy in the elderly.

## Additional file

Additional file 1: Figure S1. The gating strategy for CD4^+^, CD8^+^ T cells, B cells and NK cells, and their subsets. **Figure S2.** The population change of CD4^+^ T cells with age. **Figure S3.** The population change of CD8^+^ T cells with age. **Figure S4.** The population change of B cells with age. **Figure S5.** The population change of NK cells with age. **Table S1.** Demographics of the study subjects at the first evaluation. **Table S2.** Measures of selected biomarkers. (DOCX 6035 kb)
