# Supplementary material for: Changes in blood lymphocyte numbers with age in vivo and their association with the levels of cytokines/cytokine receptors
Source: Immun Ageing. 2016 Aug 18;13:24. doi: 10.1186/s12979-016-0079-7 (PMC4990976; doi:10.1186/s12979-016-0079-7)
Supplement: Additional file 1: Figure S1. — The gating strategy for CD4+, CD8+ T cells, B cells and NK cells, and their subsets. Figure S2. The population change of CD4+ T cells with age. Figure S3. The population change of CD8+ T cells with age. Figure S4. The population change of B cells with age. Figure S5. The population change of NK cells with age. Table S1. Demographics of the study subjects at the first evaluation. Table S2. Measures of selected biomarkers. (DOCX 6035 kb) [file 12979_2016_79_MOESM1_ESM.docx]

**Changes of blood lymphocyte numbers with age in vivo and their association with the levels of cytokines and cytokine receptors**

Yun Lin^1‡^, Jiewan Kim^1‡^, E. Jeffrey Metter^2^, Huy Nguyen^1^, Thai Truong^1^, Luigi Ferrucci^2^, and Nan-ping Weng^1*^

^1^ Laboratory of Molecular Biology and Immunology, ^2^ Translational Gerontology Branch, National Institute on Aging, National Institutes of Health, Baltimore, Maryland, 21224,USA.

**Supplemental materials**

**Fig. S1** The gating strategy for CD4^+^, CD8^+^ T cells, B cells and NK cells.

**Fig. S2** Change of CD4^+^ T cell and its subsets in blood with age.

**Fig. S3** Change of CD8^+^ T cells and its subsets in blood with age.

**Fig. S4** Change of B cells and its subsets in blood with age.

**Fig. S5** Change of NK cells in blood with age.

**Table S1**. Demographics of the study subjects at the first evaluation.

**Table S2**. List of the biomarkers.

Fig. S1. The gating strategy for CD4^+^, CD8^+^ T cells, B cells and NK cells. (A) CD4^+^ and CD8^+^ T cell gating were followed by CD28 and CD45RA gating to define the each naïve (CD4^+^CD28^+^CD45RA^+^ or CD8^+^CD28^+^CD45RA^+^) and memory (CD4^+^CD28^-^CD45RA^+^ or CD8^+^CD28^-^CD45RA^+^). From the CD4^+^ gate, CD4^+^ Treg cells are gated based on FOXP3^+^. (B) CD19^+^ B cells were further gated for naïve B cells (CD19^+^IgM^+^) and memory B cells (CD19^+^ CD27^+^). (C) NK cell gating strategy was set as CD16^+^CD14^-^.

Fig. S2. Change of CD4^+^ T cell and its subsets in blood with age. (A) Total CD4^+^ T cell numbers (cells/mL) are decreased with age in study subjects. (B) Naïve CD4^+^ T cell numbers are also decreased with age. (C) Change of Treg cell numbers with age. (D) Increase of CD4^+^CD28^-^ T cell numbers with age. Data of both first and second visit were plotted and the black line shows linear regression from all data and represents tendency through ages.

Fig. S3. Change of CD8^+^ T cell and its subsets in blood with age. (A) Total CD8^+^ T cell numbers are decreased with age in study subjects. (B) Naïve CD8^+^ T cell numbers are decreased with age. (C) CD8^+^CD28^-^ T cell numbers increase with age. Data of both first and second visit were plotted and the black line shows linear regression from all data and represents tendency through ages.

Fig. S4. Changes of B cell and its subsets in blood with age. (A) Total B cell population numbers (cells/mL) decreased with age study subjects. (B) Naïve B cell numbers decreased with age. (C) Memory B cell numbers increased with age. Data of both first and second visit were plotted and the black line shows linear regression from all data and represents tendency through ages.

Fig. S5. Change of NK cells in blood with age. NK cell numbers (cells/mL) increased with age in study subjects. Data of both first and second visit were plotted and the black line shows linear regression from all data and represents tendency through ages.

| Table S1 Demographics of the study subjects at First Evaluation^*^ | | | |
| --- | --- | --- | --- |
|  | Mean (SD) | Median | Range |
| Sample size | 223 |  |  |
| Age (average) | 68.5 (19.0) | 77 | 22-91 |
| Female (%) | 110 (49.3) |  |  |
| Education (yrs) | 17.0 (2.5) | 17 | 12-25 |
| Race (%) | Caucasian (80) |  |  |
|  | African American (14) |  |  |
|  | Other (6) |  |  |
| Smoking (%) | Current (2) |  |  |
|  | Former (39) |  |  |
|  | Never (59) |  |  |
| Cancer history % (n) | 14 (32)^**^ |  |  |
| Diabetes Mellitus % (n) | 20 (42) |  |  |
| Cardiovascular disease |  |  |  |
| ^*^ Sample demographic characteristics are presented as means with standard deviations, medians and | | | |
| ranges for the continuous variables and percentages for the categorical variables. | | | |
| ^**^ The average time between the diagnosis and blood collection was 7.9 ± 13.4 year. | | | |

| Table S2 Measures of selected biomarkers | |  |  |
| --- | --- | --- | --- |
|  | Mean (SD) | Median | Range |
| BMI | 26.9 (4.5) | 26.4 | 17.4-43.3 |
| Body fat (%) | 33.4 (8.7) | 33.6 | 9.2-53.0 |
| Diastolic Blood Pressure (mmHg) | 73.6 (10.9) | 72 | 49.0-104.0 |
| Systolic Blood Pressure (mmHg) | 131.2 (19.6) | 130 | 92.0-190.0 |
| Pulse Pressure (mmHg) | 57.6 (15.8) | 58 | 26.0-96.0 |
| Triglyceride (moles/L) | 1.1 (0.60) | 0.97 | 0.21-3.36 |
| Cholesterol (mmole/L) | 5.02 (0.97) | 4.97 | 2.79-10.81 |
| HDL (moles/L) | 1.43 (0.37) | 1.4 | 0.70-2.66 |
| LDL (moles/L) | 3.05 (0.84) | 3.01 | 1.09-7.55 |
| Fasting blood glucose (moles/L) | 5.04 (0.92) | 4.83 | 3.55-11.60 |
| IL-6 (pg/mL) | 2.36 (1.36) | 2.15 | 0.70-5.61 |
| IL6SR (pg/mL) | 36035 (9607) | 37576 | 22358-57228 |
| IL18 (pg/mL) | 279 (99) | 264 | 99-480 |
| IL1RA (pg/mL) | 83.4 (212.2) | 29.1 | 2.1-1045 |
| TNFA (pg/mL) | 9.6 (4.4) | 12.1 | 4.5-12.1 |
| IL15 (pg/mL) | 15.7 (41.5) | 3.1 | 0.81-199 |
| TNF_RII (pg/mL) | 3646 (882) | 3488 | 1996-6054 |
| TNF_RI (pg/mL) | 1847 (514) | 1728 | 1103-3406 |
| CMV IgG (U/mL) | 15.2 (15.6) | 11.4 | 0-63 |
